# Utility of a Hydrolysate from Overproduced *Paralichthys olivaceus* for Hypertension Treatment: Correlation between Physical Properties and Potent Anti-Hypertensive Activities

**DOI:** 10.3390/md20060346

**Published:** 2022-05-25

**Authors:** Hyo-Geun Lee, Jae-Young Oh, Dong-Min Chung, Min-Young Seo, Shin-Jae Park, You-Jin Jeon, Bo-Mi Ryu

**Affiliations:** 1 Department of Marine Life Science, Jeju National University, Jeju 63243, Korea; hyogeunlee92@gmail.com (H.-G.L.); youjinj@jejunu.ac.kr (Y.-J.J.); 2 Food Safety and Processing Research Division, National Institute of Fisheries Science, Busan 46083, Korea; ojy0724@korea.kr; 3 Shinwoo Corporation. Ltd. 991, Worasan-ro, Munsan-eup, Jinju 52839, Korea; jdm@shinwoocorp.com (D.-M.C.); min086@shinwoocorp.com (M.-Y.S.); sjpark@shinwoocorp.com (S.-J.P.)

**Keywords:** *Paralichthys olivaceus*, enzyme-assisted hydrolysis, spontaneously hypertensive rat

## Abstract

Aquacultured fish are the richest natural source of protein. However, their overproduced biomass is often discarded due to production imbalance, causing considerable losses to the fishery industry. Therefore, it is necessary to utilize surplus fish and add value to overproduced fish. We performed complex enzyme-assisted hydrolysis to determine the correlation between its physical characteristics and anti-hypertensive activity in vitro and in vivo using an SHR model. Protamex-Pepsin assisted hydrolysate from *Paralichthys olivaceus* (PO_pp_H) produced by complex enzyme-assisted hydrolysis contained low-molecular-weight peptides and amino acids with anti-hypertensive activity. PO_pp_H regulated blood pressure and serum angiotensin II and angiotensin-I-converting enzyme levels, and histological and ultrasound image analysis revealed substantially reduced thickness and diameter of the carotid aorta in the PO_pp_H-administered SHR group. Therefore, we propose to reduce food loss due to overproduction by utilizing the anti-hypertensive activity and physical properties of PO_pp_H; the results demonstrate its application as a therapeutic agent.

## 1. Introduction

Worldwide, approximately one-third of food produced for human consumption is lost or wasted from farm to table, amounting to around 1.3 billion tons per year [1]. In the United States, more than 35 million tons of food went into landfills in 2018 [2]. Wasted food generates severe changes in the marine and terrestrial environments. COVID-19 has exposed the vulnerabilities of food systems and heightened the need to mitigate food loss and waste, both locally and globally [3]. The Food and Agriculture Organization of the United Nations (FAO) stresses the importance of changing perceptions and providing solutions to food loss and wastage [4].

*Paralichthys olivaceus* (*P. olivaceus*) or olive flounder, belonging to the genus *Paralichthys*, is frequently found in sandy bottoms at 10–200 m. Based on the global statistics of olive flounder production, Korea is the major producer of olive flounder. In 2007, 77.6% (44,245 t) of the global olive flounder supply came from Korea. Olive flounder is often referred to as the Korean flatfish and has been the topmost aquacultured finfish in Korea over the past few decades [5]. However, the expansion of aquaculture production has created several problems, including rice reduction, high mortality due to various diseases, and a sudden increase in olive flounder production resulted in a considerable decrease in their price in the domestic aquaculture fish market. In 2019, 10,634 t of over-produced olive flounder was discarded in Jeju, Korea [6]. Earlier studies have shown that overproduction and oversupply lead to a price drop due to an imbalance of demand and supply of fish [6]. Therefore, the utilization and processing of over-produced olive flounders can increase their prices and provide a practical solution to overcome the problems associated with overproduction.

Marine fish-derived protein hydrolysates and peptides have remarkable anti-hypertensive properties [7]. Numerous studies have evaluated the effect of angiotensin-I-converting enzyme (ACE) inhibition in vitro and the anti-hypertensive mechanism underlying the lowering of blood pressure or expansion of blood vessels [8]. Our previous studies reported that a protein hydrolysate of *Paralichthys olivaceus* inhibited ACE activity and lowered systolic and diastolic blood pressure in spontaneously hypertensive rat [9]. This study focused on the biological properties of hydrolysates obtained from single enzyme-assisted hydrolysis of *Paralichthys olivaceus*. However, further studies are needed to determine the correlation between their biological activities and physical characteristics, especially the molecular weight of products obtained from single and complex enzyme-assisted hydrolysis of *Paralichthys olivaceus*.

Garcia et al. reported that products of two-stage protamex-pepsin hydrolysis had higher antioxidant, anti-hypertensive, and anti-inflammatory activities than those obtained by one-step protease hydrolysis [10]. In addition, low-molecular-weight peptides with molecular weights less than 1 kDa have greater mobility and diffusivity than high-molecular-weight molecules [11,12]. The physical and compositional characteristics of these hydrolysates are related to their functionality. In this study, we evaluated the potent anti-hypertensive activities of hydrolysates obtained from single and complex enzyme-assisted hydrolysis of *Paralichthys olivaceus* and determined the correlation between their physical characteristics and anti-hypertensive activities. In addition, by discovering the functionality of hydrolysates, we identified the utility of over-produced fish as a therapeutic agent, thus preventing food loss.

## 2. Results

### 2.1. Preparation of PO_p_H and PO_pp_H and Their ACE Inhibitory Activities

**Figure 1:**
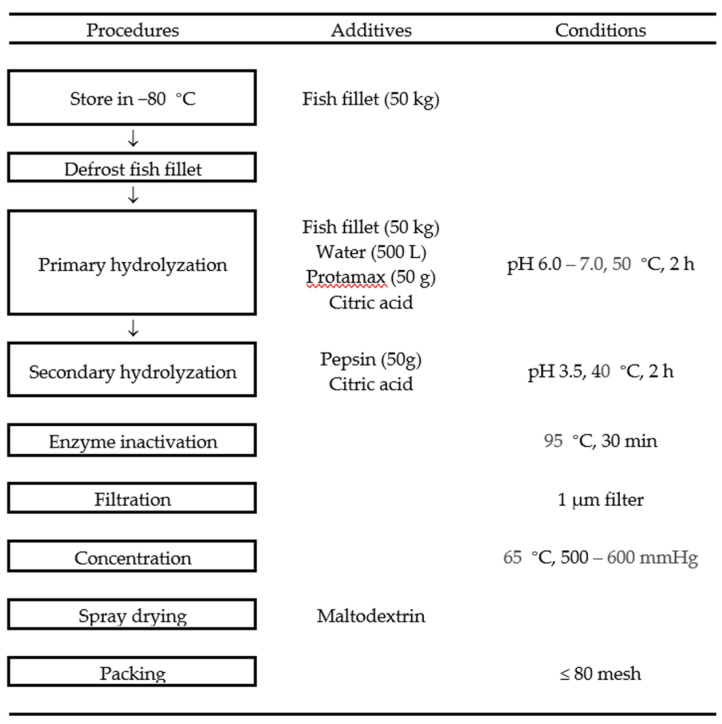
Preparation of PO_p_H and PO_pp_H.

Protamex-assisted hydrolysate from *Paralichthys olivaceus* (PO_p_H) and protamex-pepsin assisted hydrolysate from *Paralichthys olivaceus* (PO_pp_H) were prepared to establish the enzyme-assisted hydrolysis method and optimize the antihypertensive activity of PO_p_H (Figure 1). As shown in Table 1, the angiotensin-I-converting enzyme (ACE) inhibitory activity of PO_pp_H was higher than that of PO_p_H. The IC_50_ values of ACE for PO_p_H and PO_pp_H were 127.88 ± 1.32 µg/mL and 103.85 ± 0.97 µg/mL, respectively. These results suggest that ACE inhibition can be increased on the group of complex enzyme-assisted hydrolysis.

### 2.2. Physical Characterization of PO_p_H and PO_pp_H

**Figure 2:**
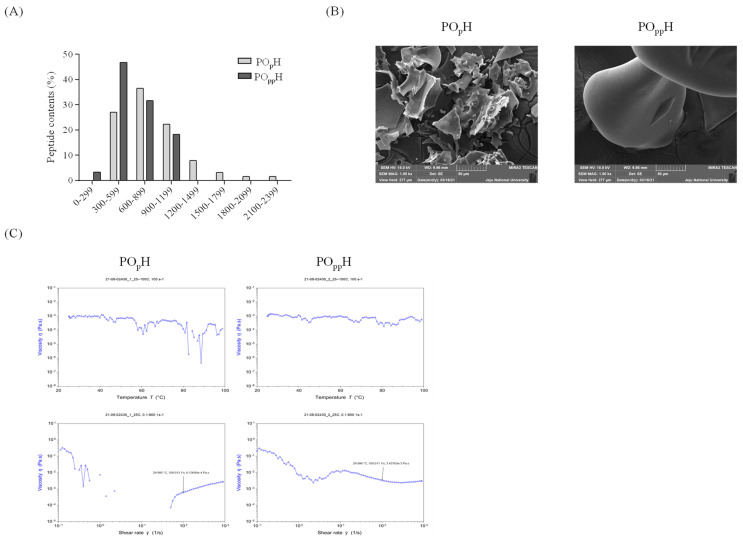
Physical characteristics of PO_p_H and PO_pp_H. (**A**) Molecular distributions, (**B**) morphological SEM images, (**C**) heat and shear activated viscosity.

The physical properties of PO_p_H and PO_pp_H were determined using LC-MS, SEM, and viscosity analysis. The molecular distributions of PO_p_H and PO_pp_H, summarized in Figure 2A, were found to be widely distributed between 300 and 2399 *m*/*z*; smaller molecular distributions were observed in PO_pp_H than PO_p_H. The molecular distributions of PO_p_H had a maximum of 300–2399 *m*/*z*, and the molecular distributions in PO_pp_H ranged from 299–1199 *m*/*z*. PO_p_H mainly had distributions in the range 600–899 *m*/*z* (36%), whereas PO_pp_H mainly had distributions in the range, 300–599 *m*/*z* (47%). The molecular mass results of PO_p_H and PO_pp_H are presented in Figure S1. The average molecular weights of PO_p_H and PO_pp_H determined using MALS revealed that they had molecular masses of $2.101\times 10^{3}$ g/mol and $8.864\times 10^{2}$ g/mol, respectively. The surface morphologies of PO_p_H and PO_pp_H were observed using field-emission SEM (FE-SEM). The SEM images are shown in Figure 2B. The SEM images of PO_p_H revealed an inconsistent surface and mainly had flattened particles with rough surfaces. A rounded and smooth surface morphology was observed for PO_pp_H under 1.00 kx magnification. The temperature- and shear-rate-dependent viscosities were analyzed for PO_p_H and PO_pp_H. As shown in Figure 2C, large differences in viscosity η values were found for PO_p_H. However, relatively small differences in viscosity η values were found for PO_pp_H. In addition, the shear-rate-dependent viscosity results revealed that the viscosity η of PO_p_H was not detected in the range, $10^{0}$−$10^{1}$ $s^{-1}$, and its values gradually increased from $10^{2}$ $s^{-1}$. The shear-rate-dependent viscosity of PO_pp_H revealed constant viscosity η values in PO_p_H. These results suggest that the temperature- and shear-rate-dependent viscosity characteristics were better maintained in PO_pp_H than in PO_p_H.

### 2.3. Amino Acid Profiles of PO_p_H and PO_pp_H

The amino acid compositions of PO_p_H and PO_pp_H were analyzed, and the results are summarized in Table 2. According to amino acid profiling, PO_p_H and PO_pp_H are composed of 18 amino acids: 12 essential amino acids (histidine; His, arginine; Arg, threonine; Thr, proline; Pro, tyrosine; Try, valine; Val, methionine; Met, isoleucine; Ile, leucine; Leu, phenylalanine; Phe, tryptophan; Trp, lysine; Lys), and 6 non-essential amino acids (cysteine, Cys; aspartic acid, Asp; glutamine, Glu; serine, Ser; glycine, Gly; alanine, and Ala). PO_p_H and PO_pp_H had high levels of Ala, Asp, Glu, Arg, Leu, Lys and all these amino acids were slightly increased with complex enzyme assisted hydrolyzation.

### 2.4. PO_pp_H Reduces SBP and DBP in the SHR Model

**Figure 3:**
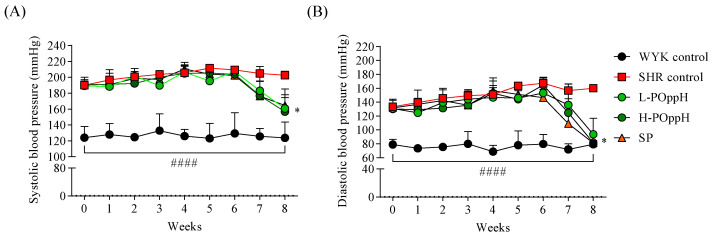
Changes in systolic and diastolic blood pressure after oral administration. (**A**) Systolic and (**B**) diastolic blood pressure. (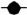) WYK control (water); (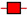) SHR control (water); (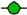) L-PO_pp_H (50 mg/kg of PO_pp_H); (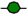) H-PO_pp_H (100 mg/kg of PO_pp_H); (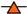) SP (50 mg/kg of SP). Data are expressed as the mean ± standard deviation (SD), (*n* = 4) in each group. Significant differences were identified at * *p* < 0.05 as compared to the SHR and #### *p* < 0.0001 as compared to WYK control.

The changes in SBP and DBP were evaluated as a measure of the antihypertensive activity of PO_pp_H during the eight-week experimental period. The initial (week 0) average values of SBP (187.38 ± 12.14 mmHg, *n* = 8) and DPB (126.92 ± 13.07 mmHg, *n* = 8) revealed the rats had hypertension at the beginning of the in vivo study. The blood pressure results presented in Figure 3 reveal that SBP and DBP were considerably downregulated from week 7 to 8 in the groups treated with low and high concentrations of PO_pp_H compared with those of the SHR groups. L-PO_pp_H decreased SBP (160.75 ± 13.82 mmHg) and DBP (93.79 ± 23.10 mmHg), and H-PO_pp_H lowered SBP (156.82 ± 28.34 mmHg) and DBP (81.69 ± 12.25 mmHg) at week 8.

### 2.5. Effect of PO_pp_H on Rat Blood Serum Biochemical Indices

To evaluate the antihypertensive effect of PO_pp_H, blood serum angiotensin II (ANG II) and angiotensin-I-converting enzyme (ACE) levels were analyzed to confirm inhibitory effect of PO_pp_H on blood ANG II and ACE (Table 3). The ANG II level was remarkably lowered in the L-PO_pp_H (1729.47 ± 429.05 pg/mL) and H-PO_pp_H (1445.30 ± 253.30 pg/mL) groups. In addition, ACE levels significantly declined in the H-PO_pp_H group compared with that in the SHR group. ACE levels considerably decreased to 8.40 ± 0.78 ng/mL in the H-PO_pp_H group.

### 2.6. Measurement of the Thickness and Ultrasound Imaging of the Carotid Aorta

**Figure 4:**
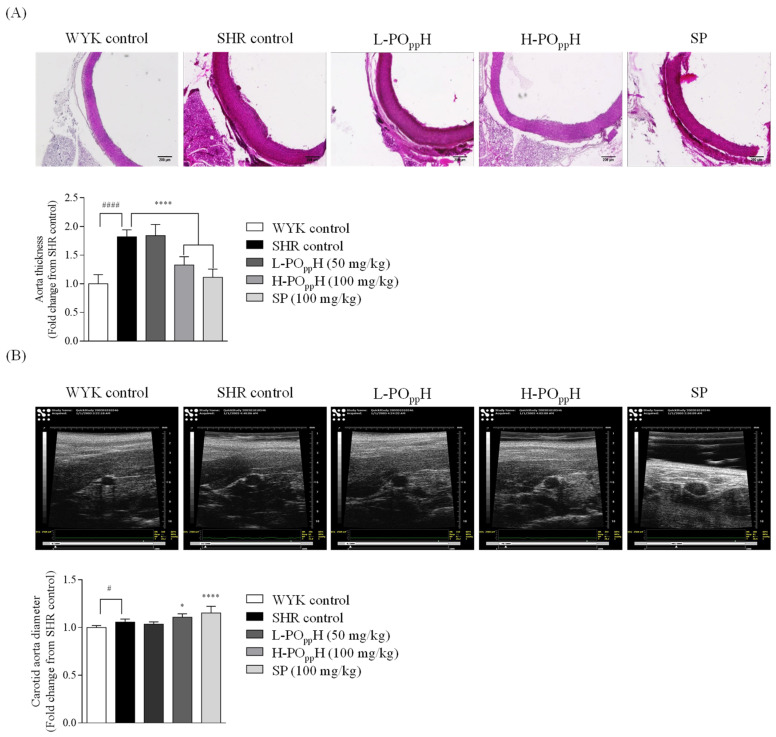
Histologic and ultrasound graphic analysis of the aorta in SHRs. (**A**) H&E staining images and (**B**) ultrasound graphic images. Data are expressed as the mean ± standard deviation (SD), (*n* = 3) in each group. Significant differences were identified at * *p* < 0.05 and **** *p* < 0.0001, as compared to the SHR control, and # *p* < 0.05 and #### *p* < 0.0001, as compared to WYK control.

The effect of PO_pp_H on the cross-sectional area of the aorta was observed using H&E staining. As shown in Figure 4A, the H&E results revealed a thicker aorta in the SHR group than in the WKY group. However, the thickness of the aorta was significantly reduced in SHRs in the PO_pp_H groups. In particular, the H-PO_pp_H group had markedly reduced aorta thickness by 1.38 ± 0.15-fold relative to the SHR group (1.82 ± 0.12-fold, ** *p* < 0.001). To determine the diameters of the carotid aorta, ultrasound observation was performed by modifying a method by Jin et al. [13]. As shown in Figure 4B, the carotid aorta images revealed a significantly increase in carotid aorta diameter of H-PO_pp_H treated group by 1.11 ± 0.03-fold relative to the SHR group (1.06 ± 0.03-fold, **** *p* < 0.0001).

## 3. Discussion

Hypertension is one of the main mediators of cardiovascular diseases [14]. Elevated central blood flow is highly burdensome and induces damage in tissues and organs, including the heart, kidney, brain, and blood vessels, ultimately resulting in organ dysfunction and failure [15]. Katz et al. reported that most patients with end-organ injuries showed a high rate of chronic and acute hypertension [16]. Therefore, the management and initial control of blood pressure (BP) could help minimize the risk of outbreak of hypertension. Recent research with animals revealed that marine fish hydrolysate and its bioactive peptide have strong angiotensin-I-converting enzyme (ACE) inhibitory activity in vitro and exponentially ameliorate blood pressure [9,17,18,19,20]. However, most studies on marine fish hydrolysate have focused on their biological properties and adopted single-enzyme-assisted hydrolysis to collect fish hydrolysates. Furthermore, the relative biological activities of single and complex enzyme-assisted hydrolysates and the subsequent changes in physical characteristics have not been fully investigated. Here, protamex hydrolysis was adopted as a one-step hydrolysis, and two-step protamex-pepsin hydrolysis was performed on fish fillets from *Paralichthys olivaceus* to assess their potent antihypertensive activities depending on changes in their physical characteristics.

The molecular distribution results revealed the presence of relatively low molecular weight peptide in PO_pp_H compared to that in PO_p_H, implying that the low molecular weight peptide was concentrated in PO_pp_H during the two-step hydrolysis. Further, the average molecular weights of PO_p_H and PO_pp_H were $2.101\times 10^{3}\;$g/mol and $8.864\times 10^{2}$ g/mol, respectively. Lin et al. reported the increased potency of ACE inhibitory activity and the potential of antihypertensive properties of low molecular weight protein hydrolysates [21]. Morphologic images of PO_p_H and PO_pp_H revealed that the surface morphologies changed during complex enzyme-assisted hydrolysis. The surface of PO_p_H showed comparatively irregular patterns and rough surface particles. However, a smooth and rounded surface was observed for PO_pp_H. The temperature- and shear-rate-dependent viscosity results indicated that the physical characteristics, especially temperature- and shear-rate-dependent viscosity, were highly maintained in PO_pp_H compared to those in PO_p_H. The amino acid compositions of PO_p_H and PO_pp_H showed an increase in the Ala, Asp, Glu, Arg, Leu, Lys in PO_pp_H relative to that in PO_p_H. These results correspond with those of previously published reports on the ACE inhibitory activity of marine fish-derived peptides [9,22,23]. Moreover, the PO_pp_H contained ACE inhibitory peptides, including Ala and Leu, indicating that PO_pp_H might have potential antihypertensive properties [24]. Lee et al. reported that the ACE inhibitory activity is closely associated with the degree of enzyme hydrolysis and peptide sequences and their amino acid composition [25]. The physical analysis results indicated that the physical and chemical characteristics changed with two-step hydrolysis. In particular, the low-molecular-weight peptides and antihypertensive amino acids were found to be concentrated by two-step hydrolysis. In addition, PO_pp_H maintained a constant viscosity under temperature- and shear-rate-dependent conditions. These results suggest that compared with single enzyme-assisted hydrolysis, the complex enzyme-assisted hydrolysis markedly increased the antihypertensive potential by increasing the low molecular peptide and antihypertensive amino acid content.

The in vitro ACE inhibitory activities revealed that ACE inhibition was significantly increased in PO_pp_H (IC_50_, 0.43 ± 0.03 mg/mL). Earlier reports by Ko et al. (2016) indicate that the pepsin-assisted hydrolysate from flounder fish showed 50% of ACE inhibition at 1.26 ± 0.14 mg/mL. These results demonstrated the ACE inhibitory activity was increased through the two-step protamex-pepsin enzyme-assisted hydrolysis, thereby influencing the selection of PO_pp_H for further in vivo animal antihypertensive studies. In vivo, hypertension was successfully induced in the SHR model (SBP: 187.38 ± 12.14 mmHg, DBP: 126.92 ± 13.07 mmHg). WKY rats maintained an SBP of 123.99 ± 14.13 mmHg and a DBP of 79.14 ± 7.20 mmHg during the initial steps of the experiment. During the eight weeks of SBP and DBP monitoring, high SBP and DBP was maintained in the SHR and PO_pp_H groups from weeks 0 to 6. However, SBP and DBP significantly decreased from week 7 in the H-PO_pp_H group compared to those in the SHR control group. However, the dose dependent SBP and DBP lowering effect of PO_pp_H on the SHR model could not be found. Nonetheless, our findings indicate that the critical concentrations of PO_pp_H on SBP and DBP were 100–200 mg/kg. Based on blood serum analysis, serum angiotensin II (ANG II) and ACE levels were significantly decreased in the PO_pp_H groups. These results correspond with those of previous reports on ANG II and ACE activation [26,27]. Chappell reported the functions of ANG II and ACE on vasorelaxation in humans [28]. PO_pp_H was found to significantly lower SBP and DBP by regulating serum ANG II and ACE levels. Histological analysis indicated that the thickness of the aorta was markedly reduced following H-PO_pp_H administration. These results correspond with those of Ashkan et al., who demonstrated the relationship between aortic wall thickness and aortic distensibility [29]. Overall, our findings suggest that H-PO_pp_H could ameliorate hypertension-induced aorta or blood vessel hypertrophy in SHRs. Moreover, ultrasound image analysis demonstrated that supplementation with PO_pp_H remarkably increased the carotid aorta diameter. Collectively, these results imply that the oral administration of PO_pp_H significantly reduced SBP and DBP by regulating ANG II and ACE levels. Furthermore, the oral administration of PO_pp_H can reduce the risk of aortic and cardiac hypertrophy.

## 4. Materials and Methods

### 4.1. Materials and Chemicals

Commercial protamex was purchased from Novo Co. (Novo Nordisk, Bagsvaerd, Denmark). Pepsin was purchased from Chongqing Jiangxia Biochemistry Pharmaceutical Co., Ltd. (Chongqing, China). The in vitro ACE kit-WST was purchased from Dojindo Inc. (Kumamoto, Japan). Serum angiotensin II (ANG II) and angiotensin-I-converting enzyme (ACE) analysis kits were purchased from LS Bio (Washington, DC, USA). All chemicals and reagents were of analytical grade.

### 4.2. Preparation of Enzymatic Hydrolysate from Paralichthys olivaceus

*Paralichthys olivaceus* were obtained from a local fish farm on Jeju Island, Korea. The fish were filleted, washed with tap water, and stored at −80 °C. The frozen fish fillet was defrosted, and 50 kg of fish fillet was hydrolyzed with protamex (50 g) for 2 h under optimal conditions (pH 6.00–7.00, 50 °C). After protamex-assisted hydrolysis, the pH of hydrolysate of *Paralichthys olivaceus* (PO_p_H) was adjusted to 3.50 with citric acid and additional 50 g of pepsin was added. The pepsin-assisted hydrolysis was continued for 2 h under 40 °C, after which, the protamex-pepsin were inactivated at 95 °C for 30 min. The mixtures were subsequently filtered (pore size: 1 µm), and the filtrate was concentrated in a vacuum concentrator (60 °C, 500–600 mmHg) up to 20 brix. The concentrated solutions were then mixed with maltodextrin and spray-dried under optimal conditions (inlet: 165–180, outlet: 70–90, rpm: 10,000). Thereafter, the spray-dried samples were stored in a freezer at −20 °C before use. Finally, the resulting protamex-pepsin assisted hydrolysate of *Paralichthys olivaceus* was named as PO_pp_H (Lot No. SW1K11SA).

### 4.3. ACE Inhibitory Activity

The ACE inhibitory effect of PO_p_H and PO_pp_H was determined using a commercial ACE assay kit (Dojindo Molecular Technologies, Inc., Kumamoto, Japan), according to the manufacturer’s instructions.

### 4.4. Molecular Distribution Based on Liquid Chromatography-Mass Spectrometry (LC-MS)

LC-MS analysis was performed to derive the molecular weight distributions of PO_p_H and PO_pp_H. The mass spectra were acquired using an UltiMate 3000 system (Dionex, Sunnyvale, CA, USA) coupled with a microQ-TOF III mass spectrometer (Bruker Corporation, 255748, Bremen, Germany). ZORBAX 300SB-C18 (1.0 × 150 mm, 3.5 µm, Agilent) was used as the separation column. The tested samples were directly infused into positive-mode ESI sources at a speed of 100 µL/min. The MS scan range was 200–2000 *m*/*z*, and the MS parameters were as follows: capillary voltage, 4500 V; dry temperature: 180 °C; funnel 1RF, 400; funnel 2RF, 400; ISCID energy, 0 eV; Hexapole RF, 250; Ion Energy, 5.0 eV; Low Mass, 300 *m*/*z*; Collision Energy, 7 eV; Collision RF, 600; Transfer Time, 80 µs; and Pre Puls Storage, 10 µs). To measure the molecular weight distributions, distilled water with 0.2% formic acid was used as the mobile phase (A), while acetonitrile containing 0.2% formic acid was used as the stationary phase (B). The tested samples were eluted using a gradient of mobile phase. The tested samples were eluted using a gradient of mobile phase (A) and stationary phase (B) at a flow rate of 100 µL/min, and the wavelength of detection was 280 nm. The following gradient elution program was employed: 0–4 min, 95:95–5:5 *v*/*v*; 4–5 min, 95:90–5:10 *v*/*v*; 5–25 min, 90:70–10:30 *v*/*v*; 25–30 min, 70:5–30:95 *v*/*v*; 30–40 min, 5:5–95:95 *v*/*v*; and 40–46 min, 5:95–95:5 *v*/*v*.

### 4.5. Determining Average Molecular Weight by Multi-Angle Light Scattering (MALS) 

To determine the average molecular weight, MALS analysis was performed using DAWN Heleos II multi-angle light scattering coupled with a Shimadzu HPLC system connected to a PL aquagel-OH MIXED-H (7.5 × 300 mm, Agilent Technologies, Santa Clara, CA, USA). The analytical sample was dissolved in 500 mM NaCl and filtered through a membrane filter (pore size: 0.22 µm). The filtered samples were subsequently loaded and eluted with 0.5 mol/L NaCl at a flow rate of 0.5 mL/min. The MALS data were analyzed using the ASTRA 6 software (Wyatt Technologies, Santa Barbara, CA, USA). 

### 4.6. Rheometry

A rotational rheometer (ARES-G2, TA Instruments Ltd., Newcastle, DE, USA) was used to assess the temperature–shear rate dependent viscosity of PO_p_H and PO_pp_H. The temperature-dependent viscosities of PO_p_H and PO_pp_H were evaluated at 20, 40, 60, 80, and 100 °C, and the shear-rate-dependent viscosity was measured in the range of $10^{-1}$ to $10^{3}$ $s^{-1}$. The following parameters were applied: minimum transducer torque in oscillation: 0.05 N·m; minimum transducer torque in steady shear, 0.1 N·m; maximum transducer torque, 200 mN·m; transducer torque resolution, 1 nN·m; strain resolution at drive motor, 0.04; measuring geometry, 25 mm plate; and measuring Gap, 1 mm.

### 4.7. Scanning Electron Microscopy (SEM)

Surface morphologies were determined using field emission scanning electron microscopy (FE-SEM; MIRA 3 TESCAN, Brno, Czech Republic) coupled with energy dispersive X-ray spectrometry (EDS). The sample was mounted on circular aluminum stubs, coating the carbon tape. After the sample was pretreated, the stub was introduced into the FE-SEM device, and the surface morphology and structure were analyzed using FE-SEM (SEM HV: 15.0 kV, magnification: 1.00 kx).

### 4.8. Amino Acid Composition

General amino acid profiles were analyzed using an amino acid auto analyzer coupled with an HPLC system (Waters, Milford, MA, USA) equipped with a Pico-Tag reverse-phase column (3.9 × 300 mm, pore size: 4 µm). For amino acid analysis, solvent A (140 mM sodium acetate, 6%(*v*/*v*) ACN, pH 5.9) and solvent B (60%(*v*/*v*) ACN) were used as mobile phases, and gradient separation was performed at a flow rate of 1 mL/min. The amino acids were detected in a Waters 2487 UV detector at 254 nm. Data were analyzed using Waters Empower 2 software. The following gradient elution of solvent A and B was employed: 0–9 min, 100:0–86:14 *v*/*v*; 9–9.2 min, 86:14–80:20 *v*/*v*; 9.2–17.5 min, 80:20–54:46 *v*/*v*; 17.5–17.7 min, 54:46–0:100 *v*/*v*; and 17.7–21 min, 0:100–100:0 *v*/*v*.

### 4.9. Animal Studies

Thirty-two male SHRs and eight male Wistar rats (WKY) (age of rats, 5 weeks old) were purchased from a commercial vendor, Jung Ang Lab Animal Inc. (Seoul, Korea). All animals had free access to tap water and chow diet containing proteins (15.2%), lipids (2.9%), cellulose (4.1%), nitrogen-free extract (60.7%), moisture (12.1%), and mineral ash (5.0%). Rats were housed in a controlled room under optimal temperature (20–22 °C), humidity (40–60%), and a 12:12 light/dark cycle. Animals were allowed to acclimate to the environment for two weeks. Thereafter, the animals were randomly divided into five groups (*n* = 8 in each group): normal control (WYK), negative control (SHR), positive control (sardine peptide (SP)), low (L-PO_pp_H), and high (H-PO_pp_H) dosage groups. The L-PO_pp_H and H-PO_pp_H mice were orally administered 50 and 100 mg/kg of PO_pp_H once daily, respectively. The positive control groups were orally administered 100 mg/kg SP once daily, and the normal and negative control groups were administered 0.9% saline. Systolic blood pressure (SBP) and Diastolic blood pressure (DBP) were monitored weekly using a CODATM tail-cuff blood pressure system (Kent Scientific Corp., Torrington, CT, USA). All experimental rats were sacrificed to retrieve their kidney, heart, and aorta tissues for further histological experiments. The animal study was approved by the International Animal Care and Use Committee (IACUC) of Jeju National University (approval number: 2020-0025, 13 July 2020).

### 4.10. Blood Serum Profiles

Blood was collected from rat heart via a cardiac puncture using an EDTA-rinsed syringe. Subsequently, the blood was transferred into a heparin-coated blood collection tube. Blood serum was allowed to coagulate for 1 h before centrifugation (3000 rpm, 15 min, 4 °C). Thereafter, the supernatant was carefully collected and stored at −80 °C.

### 4.11. Histology

Histological analysis was performed by dissecting the rat aorta. The isolated aorta tissues were fixed in 10% formalin solution and dehydrated before embedding in paraffin. Subsequently, the blocks of paraffin-embedded aorta tissue were cut into 3-µm sections using a tissue processor machine, placed on an albumin-coated slide, and dried at 37 °C for 24 h. Thereafter, the slides were deparaffinized in xylene, stained with hematoxylin and eosin (H&E) staining, and rinsed three times with deionized water. The slides were mounted with DPX mounting solution (Sigma Chemical Co., St. Louis, MO, USA). Histologic images were obtained using Lionheart FX Automated Microscope (BioTek Instruments, Inc., Winooski, VT, USA). The thickness of rat aorta tissues was measured using ImageJ software (version 1.4).

### 4.12. Ultrasound Image Analysis

SHRs (8 weeks old) were anesthetized with diethyl ether and O2 gas through a vevo compact anesthesia system. Carotid artery images were observed using the modified methods of Phaeng et al. with a Vevo 770 small animal ultrasound imaging scanner and single-element crystal mechanical imaging transducer (RMV 704; VisualSonics Inc., Toronto, ON, Canada) [30]. The diameter of the carotid aorta was quantified using MATLAB software (Math Works Inc., Natick, MA, USA).

### 4.13. Statistical Analysis

All measurements were performed in triplicate and are presented as mean ± standard deviation (SD) using the statistical package, GraphPad Prism (Version 6; GraphPad Software Inc., San Diego, CA, USA). One-way ANOVA with Duncan’s test was used to assess differences between the groups. *p*-values in the following limits were considered significant: * *p* < 0.05, ** *p* < 0.01, *** *p* < 0.001, and **** *p* < 0.0001 compared with the negative SHR control group; and # *p* < 0.05, ## *p* < 0.01, ### *p* < 0.001, and #### *p* < 0.0001 compared with the normal WYK control group.

## 5. Conclusions

In conclusion, our findings revealed that complex enzyme-assisted hydrolysis, similar to two-step protamex-pepsin enzyme-assisted hydrolysis, successfully increased the low molecular weight peptide. Moreover, physical characteristics, such as viscosity, were highly maintained in PO_pp_H. The oral administration of PO_pp_H potentially caused SBP and DBP lowering by downregulating angiotensin II and down-regulating of angiotensin-I-converting enzyme levels. Taken together, these results indicate that PO_pp_H can be utilized as an anti-hypertensive agent. Further, this study provides a rationale for clinical studies on low-molecular-weight peptides from *Paralichthys olivaceus* used as anti-hypertensive functional food or agents. The anti-hypertensive activity of *Paralichthys olivaceus* by-products could minimize the loss of aquaculture fisheries and food waste.

## Figures and Tables

**Table 1:** ACE inhibitory activity of PO_p_H and PO_pp_H.

	PO_p_H	PO_pp_H
ACE inhibitory activity, IC_50_ value (mg/mL)	0.56 ± 0.02	0.43 ± 0.03

**Table 2:** Amino acid compositions of PO_p_H and PO_pp_H.

Amino Acid	MW of Amino Acids	Concentration (µg/100 µL)	
		PO_p_H	PO_pp_H
Cys	121.160	12.49	14.95
Asp	133.100	97.36	121.21
Glu	147.130	203.10	236.56
Ser	105.090	48.64	59.63
Gly	75.070	47.24	61.93
Ala	89.100	81.76	90.16
His	155.160	5.59	6.79
Arg	174.200	83.67	105.54
Thr	119.120	40.52	48.17
Pro	115.130	31.68	36.96
Tyr	181.190	41.09	47.90
Val	117.150	60.24	69.74
Met	149.210	23.78	25.13
Ile	131.170	56.00	63.44
Leu	131.180	93.77	108.71
Phe	165.190	37.79	44.13
Trp	204.230	18.19	14.79
Lys	146.188	85.64	103.60
Total		1068.55	1259.34

**Table 3:** Effect of PO_pp_H on serum biochemistry in SHRs.

Groups	ANG Ⅱ (pg/mL)	ACE (ng/mL)
WYK control	1823.31 ± 294.33 ^#^	8.78 ± 0.87 ^###^
SHR control	1926.94 ± 266.36	9.98 ± 0.56
L-PO_pp_H	1729.47 ± 429.05 ***	8.70 ± 0.66 **
H-PO_pp_H	1445.30 ± 253.30 ****	8.40 ± 0.78 ****
SP	1685.36 ± 374.94 ***	9.01 ± 1.79 ***

Significant differences were identified at ** *p* < 0.01, *** *p* < 0.001 and **** *p* < 0.0001, as compared to the SHR control, and ^#^ *p* < 0.05 and ^###^ *p* < 0.001, as compared to WYK control.

## Data Availability

Not applicable.

## Supplementary Materials

The following supporting information can be downloaded at: https://www.mdpi.com/article/10.3390/md20060346/s1, Figure S1: Molecular distributions of POpH and POppH.
