# Supplementary figures and images for: Utility of a Hydrolysate from Overproduced Paralichthys olivaceus for Hypertension Treatment: Correlation between Physical Properties and Potent Anti-Hypertensive Activities
Source: Mar Drugs. 2022 May 25;20(6):346. doi: 10.3390/md20060346 (PMC9228416; doi:10.3390/md20060346)

## Supplementary Materials

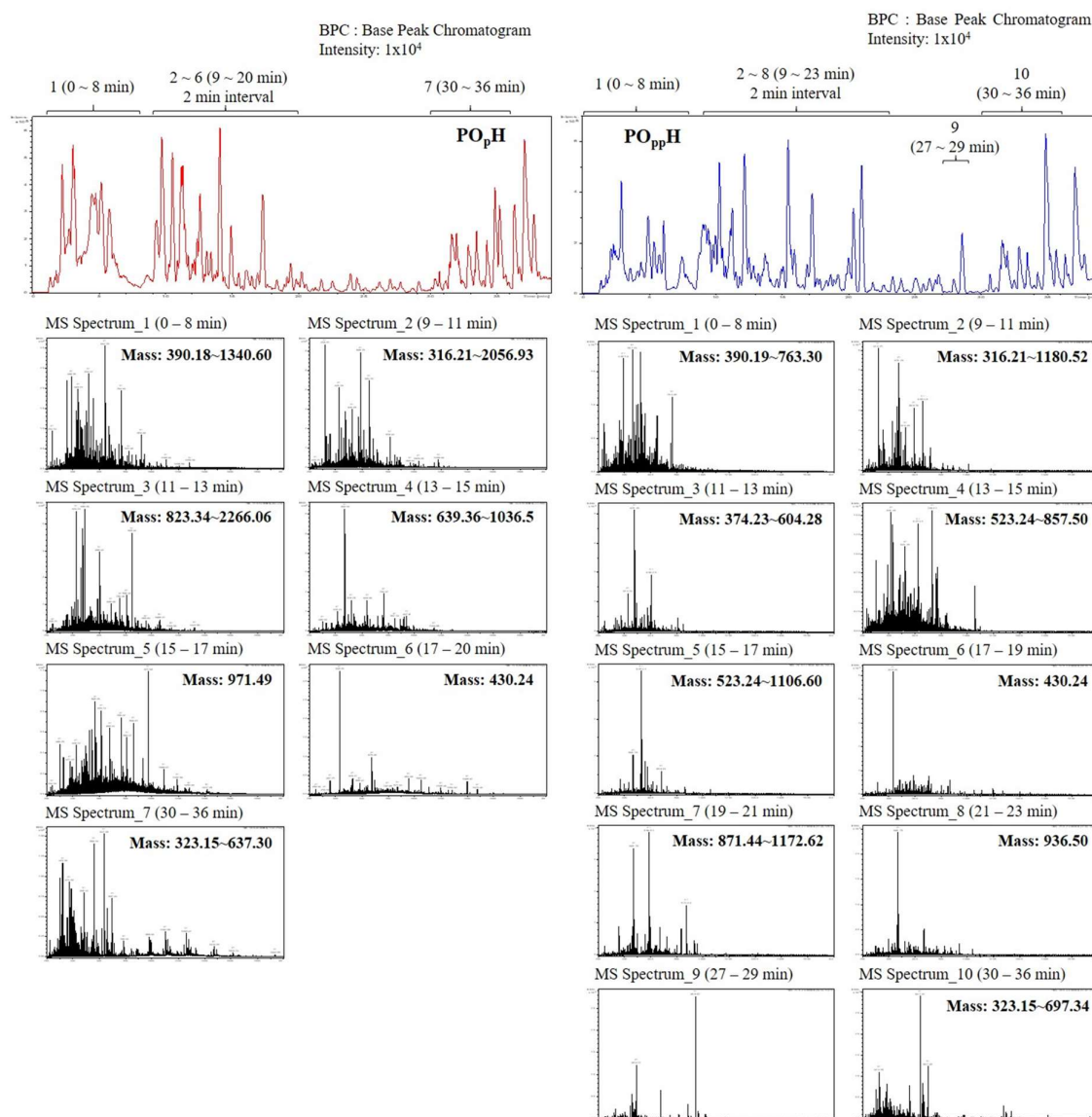

Figure S1. Molecular distributions of PO<sub>p</sub>H and PO<sub>pp</sub>H

Supplement: Supplementary file 1 [file marinedrugs-20-00346-s001.zip › marinedrugs-1725525-supplementary.pdf]
